# Severe Hepatic Enzyme Elevation and Coagulation Abnormalities in a Pregnant Woman With Recent COVID-19 Infection and Preterm Premature Rupture of Membranes: A Case Report

**DOI:** 10.7759/cureus.98462

**Published:** 2025-12-04

**Authors:** Evgenia Stagaki, Anna Damatopoulou, Nefeli Prompona, Marianna Efstratiadou

**Affiliations:** 1 Obstetrics and Gynaecology, Venizeleio General Hospital, Heraklion, GRC

**Keywords:** acute fatty liver of pregnancy, coagulopathy, covid-19, hellp syndrome, hepatic dysfunction, pregnancy complications, preterm premature rupture of membranes

## Abstract

Severe hepatic dysfunction with coagulopathy in late pregnancy is an obstetric emergency with overlapping clinical features. Acute fatty liver of pregnancy (AFLP), HELLP syndrome (hemolysis, elevated liver enzymes, and low platelets), and viral or drug-related liver injury must be considered, as delayed recognition can be fatal. Recent SARS-CoV-2 infection may further obscure the clinical picture, given its potential hepatic effects and overlap with pregnancy-related disorders. Prompt diagnosis and expedited delivery remain the cornerstone of management to prevent maternal and neonatal complications. Here, we describe a 30-year-old primigravida at 34 + 6 weeks’ gestation who presented with preterm premature rupture of membranes (PPROM) and, despite a recent mild COVID-19 illness limited to rhinitis, developed rapidly progressive hepatic enzyme elevation, renal impairment, and coagulopathy requiring urgent cesarean delivery. This case highlights the need for heightened vigilance, rapid multidisciplinary evaluation, and timely intervention when hepatic or renal function deteriorates in pregnancy.

## Introduction

Premature rupture of membranes (PROM) is defined as rupture of the fetal membranes before the onset of labor. When this occurs before 37 weeks of gestation, it is termed preterm premature rupture of membranes (PPROM). PROM affects approximately 8 % of term pregnancies, and PPROM complicates 2-3% of deliveries in the United States [1]. PROM may precipitate labor, but if labor does not begin spontaneously, the risk of complications, including chorioamnionitis, placental abruption, prematurity, and related fetal morbidity, rises substantially. Management of PPROM depends on gestational age and fetal status. When no contraindications exist, expectant management is recommended, including administration of broad-spectrum antibiotics to prolong latency, a single course of antenatal corticosteroids, and screening/prophylaxis for group B streptococci (GBS) [2].

Although infection is the most common maternal complication of PPROM, the sudden onset of marked hepatic dysfunction and coagulopathy is rare and greatly broadens the differential diagnosis. In pregnancy, the principal causes of fulminant hepatic failure are acute fatty liver of pregnancy (AFLP) and HELLP syndrome (hemolysis, elevated liver enzymes, and low platelets) [3].

AFLP is an uncommon but life-threatening disorder, affecting roughly one in 7,000-20,000 pregnancies, usually in the third trimester. It is characterized by coagulopathy, electrolyte imbalance, and multi-organ failure. Diagnosis relies on the Swansea criteria, which require ≥6 of the following in the absence of another cause: vomiting, encephalopathy, polydipsia/polyuria, abdominal pain, bilirubin >0.8 mg/dL (14 µmol/L), hypoglycemia <72 mg/dL (4 mmol/L), leukocytosis >11,000/µL, spartate transaminase (AST) or alanine aminotransferase (ALT) >42 IU/L, ammonia >47 µmol/L, uric acid >5.7 mg/dL (340 µmol/L), acute kidney injury or creatinine >1.7 mg/dL (150 µmol/L), prothrombin time >14 s, ascites or a bright liver on ultrasound, and microvesicular steatosis on biopsy [4].

HELLP syndrome, with a prevalence of 0.5-0.9 %, also typically occurs in the third trimester. Once considered a severe form of pre-eclampsia, it is now recognized as a distinct disorder because 15-20% of patients lack hypertension or proteinuria. The Tennessee classification requires all three features, i.e., hemolysis, elevated liver enzymes, and platelet count <100 × 10⁹/L, for diagnosis. Although platelet activation is common, clotting factors are usually preserved. As with AFLP, definitive treatment is prompt delivery. Notably, SARS-CoV-2 infection during pregnancy has been linked to an increased risk of HELLP syndrome [5,6].

Other important considerations include drug-induced liver injury, viral hepatitis, autoimmune hepatitis, and metabolic disorders, each with distinct management and prognostic implications. The COVID-19 pandemic adds complexity: liver enzyme elevation and coagulopathy have been reported even after mild infection, and pregnancy itself is a hypercoagulable state. However, the impact of a recent, clinically mild or resolved SARS-CoV-2 infection on obstetric liver disease remains poorly defined [7,8].

To our knowledge, this is among the first detailed reports of an acute liver-failure-like presentation in PPROM following a recent mild SARS-CoV-2 infection.

## Case presentation

A 30-year-old primigravida at 34 + 6 weeks’ gestation was transferred to our hospital for the management of PPROM. She had a clear medical history, with only allergic rhinitis and diet-controlled gestational diabetes. She denied smoking, alcohol consumption, or drug use and had no identifiable risk factors for PPROM, such as prior cervical surgery or uterine anomalies. One week before admission, she had experienced her first and only episode of SARS-CoV-2 infection, presenting with mild rhinitis only. She had completed a full COVID-19 vaccination four years earlier, with no subsequent reinfections or complications.

Before transfer, she had received a 6 mg intramuscular dose of dexamethasone for fetal lung maturation and intravenous ritodrine for tocolysis. On arrival, ritodrine was replaced with atosiban, and broad-spectrum antibiotic prophylaxis was commenced.

On admission, the patient was afebrile and normotensive, reporting headache but no abdominal pain, visual disturbance, jaundice, or oedema. Vital signs were as follows: blood pressure 118/76 mmHg, heart rate 122 beats/min, respiratory rate 20 breaths/min, oxygen saturation 98 % on room air, temperature 36.8 °C, and Glasgow Coma Scale (GCS) 15/15, with no evidence of hepatic encephalopathy.

Ultrasound demonstrated a single live fetus in cephalic presentation with an anterior grade 2 placenta, oligohydramnios, and an estimated fetal weight of 2000 g (4th percentile for gestational age); umbilical artery, middle cerebral artery, and ductus venosus Dopplers were normal. The cervix measured 18 mm with 2 cm dilation, and cardiotocography was reactive without contractions.

Initial laboratory tests showed markedly elevated hepatic transaminases and cholestatic enzymes, prolonged prothrombin and activated partial thromboplastin times, low fibrinogen, elevated creatinine and lactate dehydrogenase, and platelets at the lower end of normal. Catheterised urine demonstrated a protein-creatinine ratio of 0.5. Nephrology and cardiology consultations were requested for the evolving renal impairment and tachycardia. The bedside echocardiography and upper and lower abdominal imaging were unremarkable. Urine culture was negative, urinary sediment indicated acute tubular necrosis, and a peripheral smear showed no schistocytes.

Approximately five hours after admission the patient developed new dyspnoea, at which time her blood pressure remained stable (107/75 mmHg during nephrology review and 120/60 mmHg during cardiology review), heart rate was 148 beats/min, respiratory rate 25 breaths/min, and oxygen saturation 95 % on room air, with faint non-musical sounds at the right lung base. Repeat blood tests revealed further rises in creatinine, worsening coagulopathy, and elevated D-dimer and uric acid levels.

Because of the rapidly deteriorating hepatic and renal function, an urgent caesarean section was performed before a planned ophthalmological assessment could be completed, delivering a live male infant weighing approximately 2000 g who required neonatal intensive care. Postoperatively, the mother was admitted to the intensive care unit, where arterial blood gases showed severe metabolic lactic acidosis (lactate 8 mmol/L) with respiratory decompensation. Her liver and renal function improved steadily, and a repeat abdominal ultrasound on postoperative day 5 revealed diffuse hepatic steatosis without other abnormalities.

The patient was discharged from the ICU six days after admission, after performing an abdominal CT scan without findings, and from the hospital 12 days after with highly improved laboratory parameters. The neonate initially demonstrated elevated liver enzymes and prolonged coagulation times but recovered with supportive management. The presentation of rapidly progressive hepatic dysfunction with coagulopathy and renal impairment in late pregnancy raised concern for AFLP, atypical or normotensive HELLP syndrome, and possible COVID-19-related hepatic injury or post-viral inflammatory syndrome (Table 1).

**Table 1:** Laboratory values and progression during hospitalization WBC: white blood cell; AST: aspartate transaminase; ALT: alanine transaminase; TA-BIL: total bilirubin; D-BIL: direct bilirubin; ALP: alkaline phosphatase; γ-GT: gamma-glutamyl transferase; PT: prothrombin time; APTT:  activated partial thromboplastin time; INR: international normalized ratio; Ur: urea; Cr: creatinine; LDH: lactate dehydrogenase

	Normal range	At admission	5 hours after admission	Postoperative day 1	Postoperative day 2	Postoperative day 7	At discharge
WBC (x1000/μl)	3.8-10.5	21.2	22.9	25.7	12.6	12.3	8.7
Platelets (x10^9^/μl)	150-400	192	195	134	117	146	283
AST (U/L)	<34	396	372	276	127	161	121
ALT (U/L)	<49	847	765	512	318	202	173
TA-BIL (mg/dl)	0.3-1.2		3.8	4.1	3.9	4	2.2
D-BIL (mg/dl)	<0.3		3	3.2	3.7	2.9	1.5
ALP (U/L)	46-116		533	278	227	330	290
γ-GT (U/L)	<38	328	318	196	204	786	536
PT (sec)	10.3-13	21.6	23.8	19.5	21.2	15.2	13.5
APTT (sec)	26-38	45.8	52.4	46.8	46	35.1	33.4
INR	0.9-1.2	1.84	2.03	1.66	1.8	1.29	1.14
Fibrinogen (mg/dl)	200-400	161	142	140	120	319	392
Ur (mg/dl)	15-49.2	46	45	41	56	28	39
Cr (mg/dl)	0.5-1.2	1.78	1.95	2.02	1.93	0.75	0.72
LDH (U/L)	120-246	555	575	491	350	300	319
Uric acid (mg/dl)	3.1-6.5		11.6	11.5		2.4	2.5
D-dimers (mg/dl)	<0.5		13.08	10.24	11.14	5.18	

## Discussion

Physiological changes during pregnancy alter the liver biochemical profile, which can complicate the interpretation of abnormal results. Up to 3% of pregnancies are complicated by liver disorders. Hemodynamic changes such as increased maternal heart rate and cardiac output, together with decreased blood pressure and systemic vascular resistance, may mimic the physiology of decompensated chronic liver disease. In normal pregnancy, serum albumin falls due to plasma volume expansion, and alkaline phosphatase rises because of placental secretion. However, aminotransferases, bilirubin, and γ-glutamyl transpeptidase typically remain within normal ranges; deviations therefore warrant further investigation [9].

Severe liver disease in pregnancy is rare but carries high maternal and fetal morbidity and mortality. The most common pregnancy-specific disorders include hyperemesis gravidarum, intrahepatic cholestasis of pregnancy, pre-eclampsia/eclampsia, HELLP syndrome, and AFLP. Non-pregnancy-specific liver disease may either predate conception (e.g., viral hepatitis B/C, autoimmune hepatitis, Wilson’s disease, cirrhosis) or arise coincidentally during pregnancy (e.g., biliary disease, Budd-Chiari syndrome, drug-induced hepatotoxicity). Rapid differentiation between pregnancy-related and unrelated conditions is essential for timely management [9].

Our case represents a rare but critical situation: rapid hepatic and renal deterioration with coagulopathy in a woman who happened to present with PPROM. Importantly, PPROM itself was not the cause of the multiorgan dysfunction and is not known to precipitate hepatic failure or severe coagulopathy. It simply provided the clinical context for hospital admission, allowing early recognition of the evolving metabolic and hepatic abnormalities.

The constellation of markedly elevated liver enzymes, prolonged coagulation times, renal impairment, hyperuricemia, and leukocytosis strongly suggested AFLP or an atypical variant of HELLP syndrome. The patient met several Swansea criteria-headache, hyperuricemia, leukocytosis, coagulopathy, renal dysfunction, and transaminase elevation-making AFLP the leading diagnosis. HELLP syndrome remained a differential, but the absence of hypertension and thrombocytopenia argued against it. Nevertheless, overlap between the two disorders is well documented, and from a clinical standpoint, differentiation may be less relevant since delivery is the cornerstone of treatment [10].

The patient’s recent SARS-CoV-2 infection adds complexity. COVID-19 is now recognized as a multisystem disease with extrapulmonary manifestations, including hepatic injury. Liver involvement is reported in 14-53% of patients, with AST/ALT elevations sometimes exceeding 1,000 U/L. Bilirubin and alkaline phosphatase abnormalities are also described in up to half of cases [11]. Proposed mechanisms include direct viral cytotoxicity, systemic inflammation, hypoxia, endothelial dysfunction, and drug-induced hepatotoxicity. Although more pronounced in severe disease, even mild infections can be followed by abnormal liver function tests. Pregnant women may be particularly susceptible due to hypercoagulability, reduced hepatic reserve, and altered immune responses. Reports suggest that hepatic dysfunction in COVID-19-positive pregnancies is associated with preterm birth, cesarean delivery, and low birth weight. In our patient, the temporal proximity of a mild COVID-19 illness raises the possibility that post-viral hepatic injury acted synergistically with pregnancy-specific pathology to accelerate hepatic and renal decompensation [12].

Management focused on urgent delivery and supportive care, consistent with standard recommendations for both AFLP and HELLP, which are generally self-limiting postpartum. The rapid deterioration in renal function, coagulopathy, and lactic acidosis prompted emergency cesarean section. Multidisciplinary input, from obstetrics, nephrology, cardiology, intensive care, and neonatology, was critical to favorable maternal and neonatal outcomes. The neonate’s transient biochemical abnormalities raise the possibility of transplacental metabolic or inflammatory contributions, which warrant further investigation [13].

## Conclusions

Rapidly progressive hepatic failure-like illness in pregnancy is uncommon but potentially life-threatening. In this case, PPROM was an incidental finding and not a contributing factor to the maternal hepatic or renal deterioration. Even a mild recent COVID-19 infection may contribute to, or mimic, pregnancy-specific liver disease such as AFLP or atypical HELLP syndrome.

This case emphasises several key clinical lessons. Clinicians should maintain a broad differential diagnosis when hepatic dysfunction develops in pregnancy, even in the absence of hypertension, proteinuria, or thrombocytopenia. Serial laboratory monitoring is vital, as biochemical deterioration can occur within hours. Moreover, a recent SARS-CoV-2 infection should be considered a potential co-factor in hepatic and coagulation abnormalities during pregnancy, as post-viral immune dysregulation and endothelial injury may amplify gestational metabolic stress.

Early recognition, timely delivery, and coordinated multidisciplinary management remain the cornerstone of care to optimize maternal and neonatal outcomes.
